# Uniform Field Re-entrant Cylindrical TE$_{01\text {U}}$ Cavity for Pulse Electron Paramagnetic Resonance Spectroscopy at Q-band

**DOI:** 10.1007/s00723-017-0955-0

**Published:** 2017-09-30

**Authors:** Jason W. Sidabras, Edward J. Reijerse, Wolfgang Lubitz

**Affiliations:** Max Planck Institut für Chemische Energiekonversion, Stiftstrasse 34-36, 45470 Mülehim an der Ruhr, Germany

## Abstract

Uniform field (UF) resonators create a region-of-interest, where the sample volume receives a homogeneous microwave magnetic field ($B_1$) excitation. However, as the region-of-interest is increased, resonator efficiency is reduced. In this work, a new class of uniform field resonators is introduced: the uniform field re-entrant cylindrical TE$_{\text {01U}}$ cavity. Here, a UF cylindrical TE$_{\text {01U}}$ cavity is designed with re-entrant fins to increase the overall resonator efficiency to match the resonator efficiency maximum of a typical cylindrical TE$_{011}$ cavity. The new UF re-entrant cylindrical TE$_{\text {01U}}$ cavity is designed for Q-band (34 GHz) and is calculated to have the same electron paramagnetic resonance (EPR) signal intensity as a TE$_{011}$ cavity, a 60% increase in average resonator efficiency $\Lambda _\mathrm{ave}$ over the sample, and has a $B_1$ profile that is 79.8% uniform over the entire sample volume (98% uniform over the region-of-interest). A new H-type T-junction waveguide coupler with inductive obstacles is introduced that increases the dynamic range of a movable short coupler while reducing the frequency shift by 43% during over-coupling. The resonator assembly is fabricated and tested both on the bench and with EPR experiments. This resonator provides a template to improve EPR spectroscopy for pulse experiments at high frequencies.

## Introduction

With the introduction of uniform field resonators for electron paramagnetic resonance (EPR) spectroscopy [1–4], a cavity can be designed to have a microwave magnetic field ($B_1$) strictly uniform over a region-of-interest of any length. However, as one increases the length of the region-of-interest, the resonator efficiency is lowered due to the reduction of stored energy within the cavity volume. By extending the uniform field concept to loop-gap resonators (LGR) [5], it became possible to design resonators with both high efficiency and uniform field distributions [6].

Yet, as one designs higher frequency LGR, the sample loop diameter must be significantly reduced to lower inductance and/or the number of gaps must be increased to lower capacitance [6–8]. The sample loop diameter imposes a limit on the capillary size and sample volume, potentially limiting concentration sensitivity. This limit is not present in typical cavities, such as the cylindrical TE$_{011}$ with a capillary sample, where a broad sample volume optimum exists [9].

**Fig. 1:**
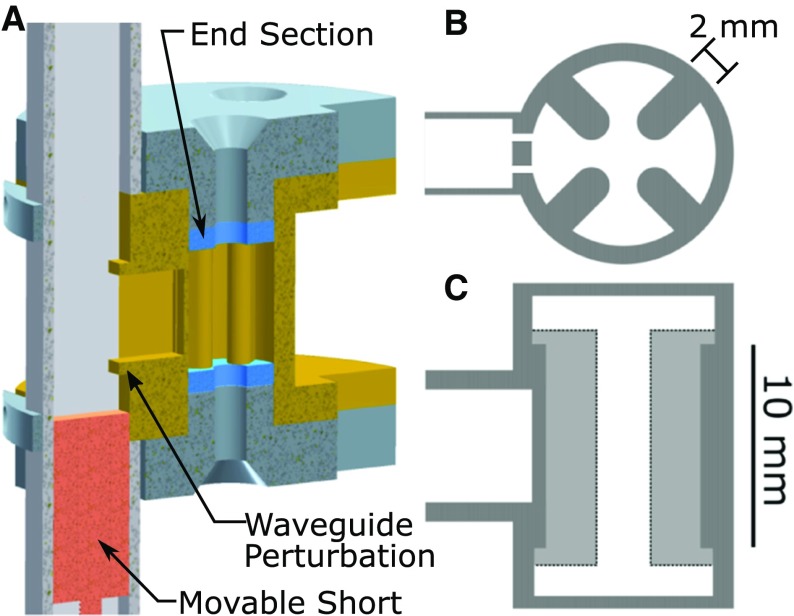
**a** Half-structure resonator assembly CAD drawing showing the brass resonator (gold), brass end plates (grey), Rexolite end sections (blue), and copper waveguide (light grey). The waveguide H-type T-junction coupler with inductive obstacles and brass movable short is also illustrated. The re-entrant geometry is further detailed in **b** the top view, showing the re-entrant fins and dual-slot iris, and **c** the side view, showing the 10 mm uniform field region-of-interest

In this work, we introduce a uniform field (UF) re-entrant cylindrical TE$_{\text {01U}}$ cavity for Q-band (34 GHz) pulse EPR spectroscopy, as illustrated in Fig. 1. Re-entrant geometries are defined as waveguide structures, where perturbations are placed in regions of large electric field to lower the cut-off frequency of the waveguide and increase the stored energy of the cavity [10, 11]. In a cylindrical waveguide, for a fixed cut-off frequency, the diameter of the waveguide is decreased as the re-entrant perturbations are extended into the electric field. If one was to make a re-entrant cavity by placing a shorted top/bottom on the waveguide and the resonant frequency is held constant, the stored energy of the cavity would increase as the re-entrant perturbations are increased. When sufficiently close, the re-entrant perturbations behave like plate capacitors. In fact, an LGR can be considered a highly re-entrant waveguide operating at cutoff. It is the geometric space between cavities and LGRs that the re-entrant geometry explores.

In the present design, four 2 mm re-entrant fins are extended into a UF cylindrical TE$_{\text {01U}}$ cavity, as shown in Fig. 1b, and the UF region-of-interest is elongated to 10 mm, as shown in Fig. 1c. This geometry provides an enhanced efficiency parameter, increased EPR signal intensity, and a uniform $B_1$ field along the sample volume.

In general, UF resonators exhibit a number of advantages compared to traditional cavities. Typically, a cavity geometry has a cosine dependence of the microwave magnetic field in the transverse *z*-direction. With a UF resonator, a region-of-interest is designed to be strictly uniform and can be extended beyond a half-wavelength. Uniform field resonators (1) provide better quantitative measurements reducing the need to calibrate the resonator $B_1$ profile [12]; (2) allow the region-of-interest to be extended to provide a larger sample volume, increasing the EPR concentration sensitivity; (3) can perform reliable continuous-wave (CW) saturation studies [13] and more reliable $T_1$ measurements using saturation recovery; (4) can be used in pulse experiments with the need for coherent pulses (such as ESEEM/HYSCORE, DEER, and ELDOR-detected NMR) and provides a more uniform $B_1$ excitation along the entire sample volume [14, 15]; and (5) provides uniform excitation for arbitrary-waveform generator (AWG)-shaped inversion pulses [16, 17] and frequency sweeps [18].

In addition, an H-type T-junction waveguide coupler with inductive obstacles is used to couple from the transmission waveguide to the resonator, as shown in Fig. 1a. The introduction of the inductive obstacles increases the dynamic range of a movable short coupler while reducing the frequency shift during matching. A dual-slot iris is employed to lower the stored energy of the iris and minimize $B_1$ perturbations along the sample volume [6].

The resonator assembly is fabricated and tested both on the bench and with EPR experiments. Experimental bench test measurements of the resonator characteristics are provided and compared to computer simulations. The $B_1$ profile is measured on the bench using the method of perturbing spheres.

## Methods

Finite-element simulations were performed on a Fujitsu workstation with dual eight-core Xeon E5-2640 2.60 GHz processors with 15 MB of L2 Cache per chip and 124 GB of system DDR4 RAM. A RAM drive was set up with 16 GB of RAM. The temporary directory and simulation files were stored in the RAM drive to reduce hard-drive bottlenecks. This system has been optimized for simulations with new versions of ANSYS (Canonsburg, PA, USA) High-Frequency Structure Simulator (HFSS; v. 18.2) and are able to take advantage of all 16 CPUs during finite-element modeling matrix solving. The operating system was Windows 7 64 bit. The eigen-mode and driven-mode solvers were used and typical simulation times were 15 min. All simulations were performed around 34 GHz.

EPR signal intensity and resonator efficiency values were calculated using ANSYS HFSS [19] and tabulated for comparison with typical resonator geometries, such as the cylindrical TE$_{011}$ cavity [20]. Two EPR signal conditions are calculated: signal unsaturable (Su) and signal saturable (Ss). In continuous-wave (CW) experiments, signal unsaturable is defined as the EPR signal intensity at constant incident power, while signal saturable is defined as the EPR signal intensity at constant $B_1$. For pulse experiments, signal saturable is proportional to the EPR signal intensity. A 2.8 mm OD and 1.8 mm ID quartz capillary (QSIL GmbH, Ilmenau, Germany) with ice sample ($\epsilon _r=3.17-i0.0035$ [21]) was used in the simulations.

To better assess the uniformity of the $B_1$ field, we define the resonator efficiency as an average over the sample volume:

1 $$\begin{aligned} \Lambda _\mathrm{ave} = \frac{\int B_\mathrm{1r} \, \mathrm{d}V}{(P_\mathrm{s} + P_\mathrm{w} + P_\mathrm{e})^{1/2} V} \quad [mT/W^{1/2}], \end{aligned}$$

where $B_\mathrm{1r}$ is the clockwise (or counter clockwise) rotational component of the linear $B_1$ field perpendicular to the static magnetic field, in millitesla, integrated over the sample volume, *V* [6]. The power loss in the system for the sample, resonator walls, and Rexolite end sections is defined as $P_s$, $P_w$, and $P_e$, respectively. The efficiency parameter $\Lambda _\mathrm{max}$, as introduced by Hyde et al. [22], is defined as

2 $$\begin{aligned} \Lambda _{\max } = \frac{\mathrm{Max}(B_\mathrm{1r})}{(P_\mathrm{s} + P_\mathrm{w} + P_\mathrm{e})^{1/2}} \quad [mT/W^{1/2}], \end{aligned}$$

where Max($B_\mathrm{1r}$) is the maximum $B_\mathrm{1r}$ in the sample (typically in the center of the cavity) and is assumed to be uniform over the sample volume. The $\Lambda _\mathrm{ave}$-to-$\Lambda _\mathrm{max}$ ratio can be used as a metric to the uniformity of the resonator [6]. In this work, we defined the $B_\mathrm{1}$ profile uniformity as

3 $$\begin{aligned} \Delta B_1 = \frac{\left| \Lambda _\mathrm{max} - \Lambda _\mathrm{ave} \right| }{\Lambda _\mathrm{max}} \times 100\%. \end{aligned}$$

After the resonator geometry is simulated, it is transferred to the 3D CAD software tool AutoDesk Inventor Professional, where the manufacturing details and geometric dimensions and tolerances are added. The model makers at the Max Planck Institutes for Chemical Energy Conversion and Kohlenforschung (Mülheim, Germany) performed the fine-mechanics tooling and die-sink electric discharge machining (EDM) manufacturing needed to fabricate the assembly. The prototype UF re-entrant cylindrical TE$_{\text {01U}}$ cavity was fabricated from brass for the resonator body and end-caps. The end sections were manufactured out of Rexolite plastic. Geometric STL files are provided at the Act-EPR website (http://www.act-epr.org).

Resonator characteristics, such as the frequency measurements, $Q_0$ value, over-coupling profiles, and sample frequency shifts, were performed on an Agilent 8722ES (now Keysight Technologies; Santa Rosa, CA, USA) vector network analyzer. A 2.8 mm OD and 1.8 mm ID quartz capillary (Vitrocom; Mountain Lakes, NJ, USA) was filled with polystyrene (PS) and a small (0.5 mm diameter) metallic probe was used as the test sample for the method of perturbing spheres. The method of perturbing spheres measures the increase in the microwave frequency as the metallic probe is stepped through the cavity volume. The size of the metallic sphere is chosen, so the overall frequency shift was less than 100 MHz.

To further test the $B_\mathrm{1}$ field uniformity, a nutation experiment was performed on a Bruker E580 spectrometer with a home-built transceiver accessory operating at Q-band with 10 W of total power. The nutation experiment consists of an initial preparation pulse of varying length ($\tau _n$), fixed delay ($t_1$ of 5000 ns), and a two-pulse detection. The pulse length $\tau _n$ was stepped by 4 ns over 2048 steps and a two-pulse detection echo was recorded [14]. The two-pulse detection echo was configured with a 60 and 120 ns pulse with a delay $t_2$ of 300 ns. The integration of the echo was recorded. The sample consisted of 0.1% $\alpha$,$\gamma$-bisdiphenylene-$\beta$-phenylallyl (BDPA) by weight in polystyrene (PS) and was placed in a 2.8 mm OD and 1.8 mm ID quartz capillary. Two samples were used. The first extended the entire length of the cylindrical TE$_{011}$ and re-entrant TE$_{\text {01U}}$ cavity length. The second was a 9.5 mm sample to place in the 10 mm region-of-interest of the UF re-entrant TE$_{\text {01U}}$ cavity. The data were background subtracted in Xepr with a first-order ($1+x$) polynomial.

## Design

### Re-entrant TE$_{\text {01U}}$ Cavity

**Fig. 2:**
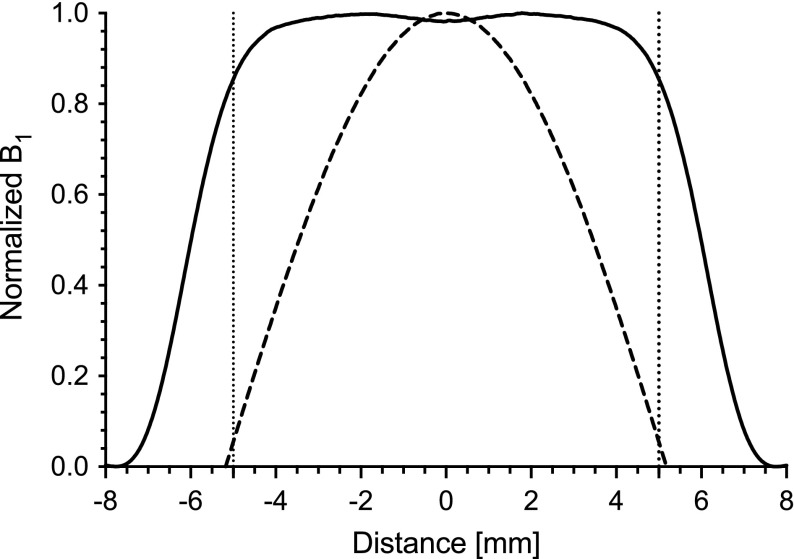
Ansys HFSS simulation showing normalized $B_1$ field of the cylindrical re-entrant TE$_{\text {01U}}$ (solid) compared to the cylindrical TE$_{\text {011}}$ cavity. Dotted lines mark the region-of-interest of the cylindrical re-entrant TE$_{\text {01U}}$ cavity

The cylindrical TE$_{011}$ cavity is a standard cavity for Q-band systems. The high $Q_0$ value and sample volume make it a good general-purpose resonator. However, for pulse experiments, the $B_1$ field variation is 50.9% over the cavity volume. The normalized $B_1$ field for a cylindrical TE$_{011}$ cavity is shown in Fig. 2 as a dashed line. In a TE$_{011}$ cavity, when a 90 or 180$^{\circ }$ pulse is applied, a significant portion of the spins in the sample volume is either over or under excited.

If one designs a UF cylindrical TE$_{\text {01U}}$ cavity with a region-of-interest of 10 mm, as described in Refs. [1, 2], the $B_1$ variation over the cavity volume is reduced to 20%. However, the overall $\Lambda _\mathrm{max}$ is reduced by 39% compared to a TE$_{011}$ cavity, due to the decrease in stored energy in the entire cavity volume. A solution to increase the resonator efficiency is to introduce re-entrant fins, as shown in Fig. 1b, where the electric field is concentrated, as shown in 3a, and results in a $\Lambda _\mathrm{max}$ decrease of 11%, but an overall $\Lambda _\mathrm{ave}$ increase of 59.6%. The calculated resonator characteristics of the proposed UF re-entrant TE$_{\text {01U}}$ cavity and comparison to a TE$_{011}$ and UF cylindrical TE$_{\text {01U}}$ cavity are found in Table 1.

**Table 1:** Ansys HFSS simulated resonator characteristics

Geometry	Cyl. TE$_{\text {011}}$ * D*/*L* = 1	UF Cyl. TE$_{\text {01U}}$	UF TE$_{\text {01U}}$ re-entrant
Frequency	34.3	34.5	34.1
*Q* $_0$-Value	13000	5900	1880
Signal, S$_u$	1	0.73	1.06
Signal, S$_s$	1	0.83	1.18
$\Lambda _\mathrm{max}$ (mT/W$^{1/2}$)	1.06	0.65	0.94
$\Lambda _\mathrm{ave}$ (mT/W$^{1/2}$)	0.52	0.52	0.83
$\Delta$B$_1$	50.9%	20%	11.7%

**Fig. 3:**
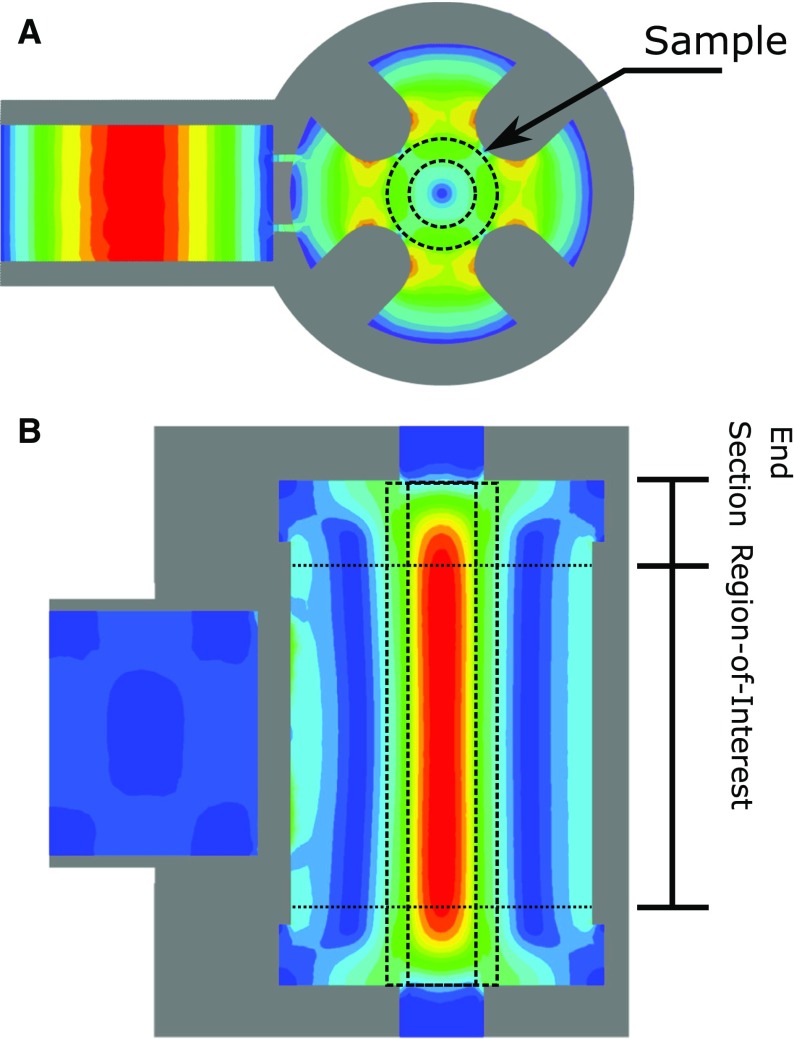
Ansys HFSS simulation showing the microwave **a** electric and **b** magnetic fields of the uniform field re-entrant cylindrical TE$_{\text {01U}}$ cavity. Each iris is 0.2 mm wide and extends over the entire waveguide length

Using Ansys HFSS, a UF re-entrant TE$_{\text {01U}}$ cavity is designed by the following procedure: (1) an eigen-mode solution of the central section with sample is simulated with a perfect magnetic field boundary condition. This provides the resonant frequency of the central section at cutoff with sample. (2) The region-of-interest is extended to 10 mm and Rexolite end sections are added to the simulation at a nominal height. (3) The end sections are varied until the eigen-frequency matches the cut-off frequency. (4) An iris is introduced and the end sections are adjusted to accommodate the frequency shift. (5) Once completed, the resonator is imported into AutoDesk Inventor and prepared for fabrication.

By properly matching the end sections, a uniform $B_1$ field can be realized, as illustrated in Fig. 3b. The normalized $B_1$ field profile is shown in Fig. 2 as a solid line. In the 10 mm region-of-interest, the $B_1$ field profile is 98% uniform.

As shown in Fig. 1c, the re-entrant fins do not extend fully into the end-section region. This design choice causes the end section to be electrically larger (shorter wavelength, $\lambda _g$) and reduces the end-section size needed to produce the matching criteria for the region-of-interest. Since the end sections are electrically larger, the roll-off is steeper compared to the re-entrant section. Decreasing the roll-off region of the resonator minimizes the sample volume that is excited by non-uniform fields.

### Dual-Slot Iris Design

A dual-slot iris was designed to couple the UF re-entrant TE$_{\text {01U}}$ cavity. The use of dual-slot irises reduces $B_1$ perturbations due to the stored energy in the iris. For UF resonators, the dual iris also reduces coupling to higher order modes that may exist because of the large length of the region-of-interest [6]. The size of a single capacitive iris needed to couple the resonator was 0.45 mm. A dual-slot iris with 0.2 mm thickness each was needed to achieve the same coupling. The geometry is shown in Fig. 1a, c and the electric field profile in Fig. 3a.

### Waveguide H-type T-junction Coupler with Inductive Obstacles

**Fig. 4:**
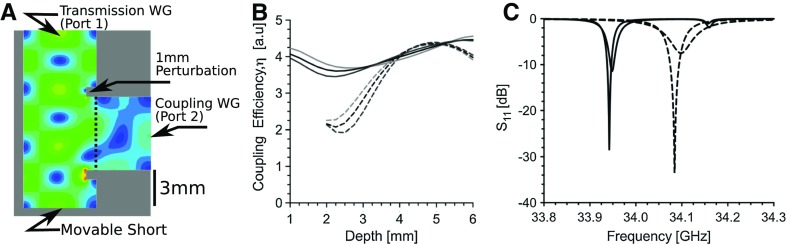
**a** Waveguide H-type T-junction coupler geometry with perturbations showing the transmission waveguide, coupling waveguide, and movable short. **b** Ansys HFSS simulations showing the coupling efficiency without (solid) and with 1 mm perturbations (dashed) for three frequencies (33.5, 34, and 34.5 GHz, light grey, black, and dark grey, respectively). **c** Simulations showing the reflection coefficient S$_{\text {11}}$ without (solid) and with 1 mm perturbations (dashed) at various depths of the movable short (coupled and over-coupled; 3.5 and 5 mm). Operating frequency is 34.09 GHz

In order for the resonator assembly to fit in a 40 mm diameter cryostat, an H-type T-junction coupler was implemented with a sliding short matching system. This sliding short is typical for a Q-band TE$_{011}$ cavity and provides a robust coupling method for room temperature to sub-10 K measurements [20]. Typically, coupling to a TE$_{011}$ cavity is performed by introducing an iris to the H-plane sidewall of the transmission waveguide. However, due to the oversized end sections, a coupling waveguide of 5.07 mm length is introduced perpendicular to the transmission waveguide, as illustrated in Fig. 4a.

Three features describe an H-Type T-junction coupler: (1) the H-type T-junction coupler is similar to the H-arm of a magic-Tee coupler [10, 11]. (2) The coupling waveguide is at least $\lambda _g$/2 in length. (3) To maximize coupling, an “inductive obstacle” the sub-wavelength H-type T-junction is introduced to the transmission waveguide. The “inductive obstacle”, described in Ref [11] as a “Window Formed by One Obstacle”, is introduced to the H-plane around the coupling waveguide and extend 1 mm into the transmission waveguide, as illustrated in Fig. 4a. This inductive obstacle creates a favorable geometry for electric field coupling and increases the coupling efficiency.

Plotted in Fig. 4b is the simulated coupling efficiency transmission can be calculated using an overlap integral [23] of the two electric fields and is defined as

4 $$\begin{aligned} \eta = \frac{\left| \int E_{t} \cdot E^*_{c}\, \mathrm{d}A\right| ^2}{\int \left| E_t\right| ^2 \mathrm{d}A \int \left| E_c\right| ^2 \mathrm{d}A}, \end{aligned}$$

where $E_{t} \cdot E^*_{c}$ represents the electric field coupling over the waveguide interface area between the electric field of the transmission waveguide ($E_{t}$) and the coupling waveguide ($E_{c}$) at the interface area A, shown as a dotted line in Fig. 4a.

The coupling efficiency between port 1 at the transmission waveguide and port 2 at the coupling waveguide is shown in Fig. 4b, where the coupling efficiency without the waveguide perturbations (solid) and with 1 mm perturbations (dashed) is plotted. Three frequencies (33.5, 34, and 34.5 GHz, light grey, black, and dark grey, respectively) are used to show the frequency dependence. Lower coupling efficiency under-couples the resonator. The resonator is critically coupled when the movable short is around 3.5 mm depth at 34 GHz and maximum over-coupling occurs at 5 mm. The waveguide H-type T-junction coupler with the perturbation illustrates a more dynamic range in coupling for the same distance and a flatter response for maximum over-coupling with a 1 GHz frequency range. To produce the same coupling range without the waveguide perturbations, the iris must be 25% larger. A larger iris leads to inhomogeneity of the $B_1$ field around the iris and in the region-of-interest.

The UF re-entrant TE$_{\text {01U}}$ cavity is designed to be over-coupled. Shown in Fig. 4c is the effect of the movable short on the reflection coefficient S$_{\text {11}}$ of the UF re-entrant TE$_{\text {01U}}$ with a range of coupling positions (3.5 and 5 mm, coupled and over-coupled). The resonator microwave frequency shift with coupling is reduced from 14.4 to 8.2 MHz using the H-type T-junction coupler with perturbations. Lower microwave frequency pulling occurs due to a reduction in the stored energy in the region of the coupler.

In addition, for the same resonator geometry, there is 145 MHz shift in operating frequency from its eigen-frequency of 34.1 GHz due to the impedance of the coupler without the perturbations. The coupler without perturbations has more stored energy and more reactance, consistent with the understanding of coupling systems with frequency dependence [24]. This causes a shift in the real part of the microwave frequency to compensate for the imaginary part of the assembly reactance and makes the assembly more frequency dependent.

## Results

The $Q_0$ value of the UF cavity was measured to be 1330 with a distilled water ice sample at a frequency of 33.95 GHz. In addition, the frequency shift of the re-entrant TE$_{\text {01U}}$ cavity as match was adjusted from critically coupled (− 45 dB ) to over-coupled (− 9 dB) was 6.96 MHz shift. Consistent with simulations, see Fig. 4c.

**Fig. 5:**
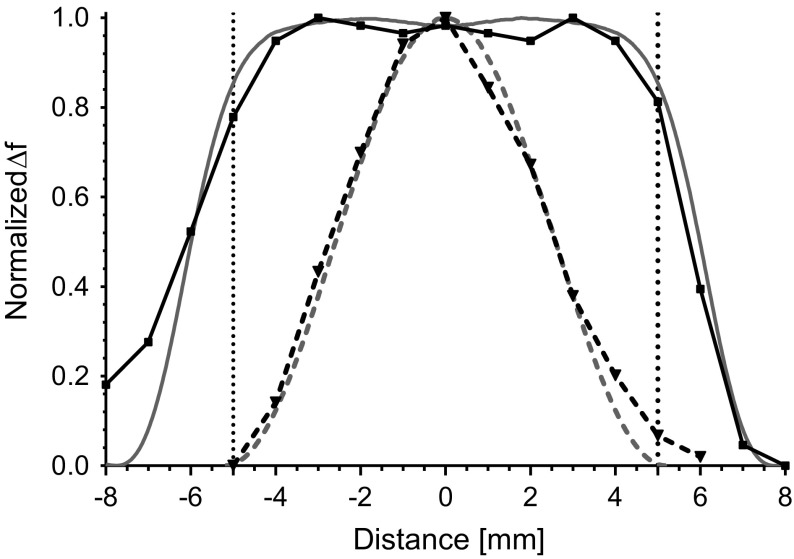
Method of perturbing spheres showing the normalized $\Delta$f along the axis of the cylindrical re-entrant TE$_{\text {01U}}$ (solid) compared to the cylindrical TE$_{\text {011}}$ cavity. Dotted lines mark the region-of-interest of the cylindrical re-entrant TE$_{\text {01U}}$ cavity. Comparison to Ansys HFSS simulations is shown in grey

The change in frequency due to the presence of a small metallic probe is shown in Fig. 5. Measurements of the UF re-entrant TE$_{\text {01U}}$ cavity are shown as a solid line and a cylindrical TE$_{011}$ are shown as a dashed line. The profiles here should be compared to the Ansys HFSS simulations in Fig. 2, repeated as grey for convenience.

**Fig. 6:**
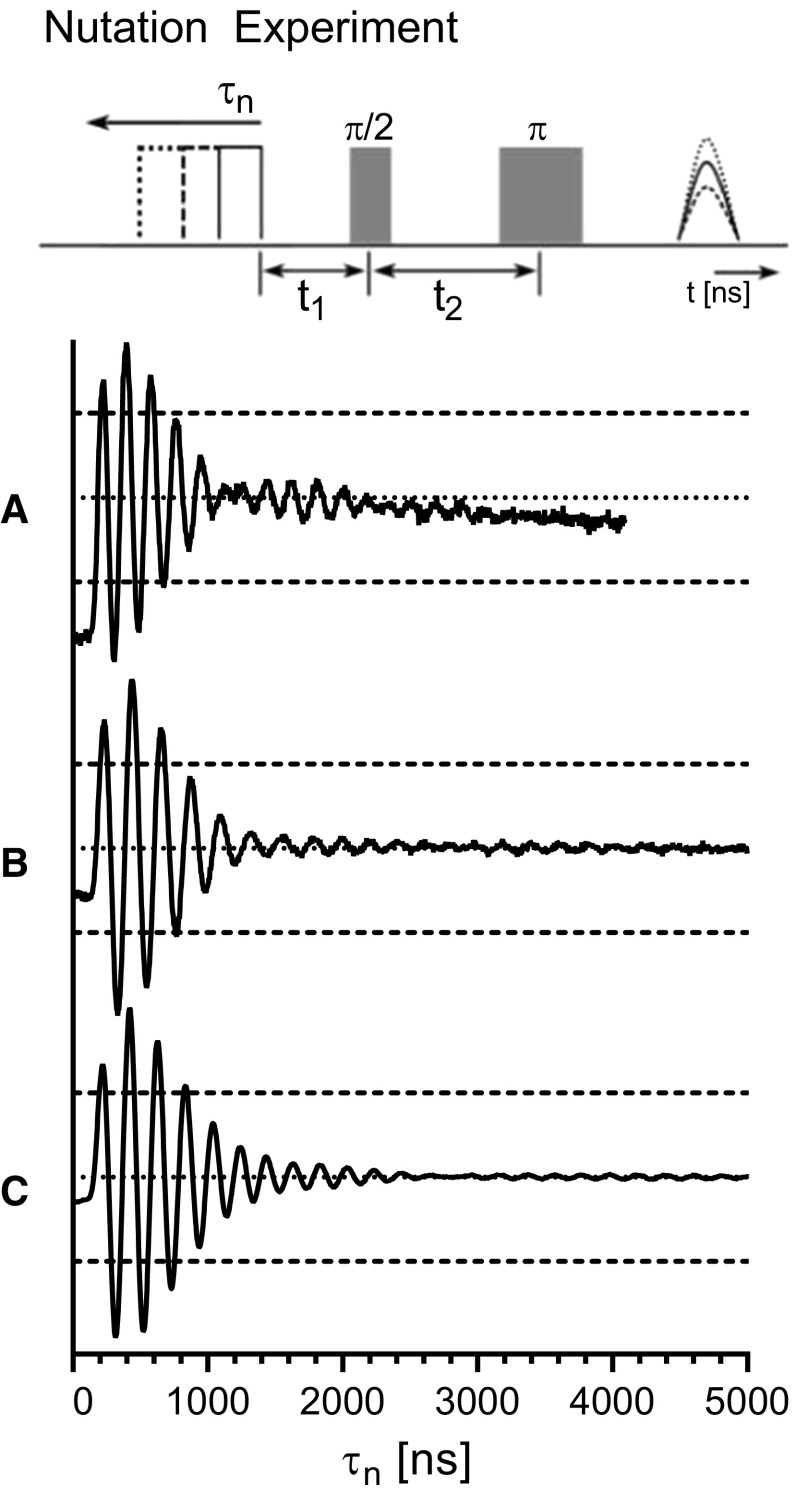
Nutation experiment on a BDPA sample performed using the **a** cylindrical TE$_{011}$ cavity (**b**) and the uniform field re-entrant TE$_{\text {01U}}$ cavity with the sample extending the entire length and **c** 9.5 mm sample centered in the region-of-interest. Pulse lengths were 120 ns for $\pi$ pulse and the preparation pulse length was stepped 4 ns. Dotted lines show the nutation center, while dashed lines show 50% signal markers

Shown in Fig. 6 are the data from the nutation experiment. Dotted lines represent the center of the nutations, while dashed lines show 50% signal markers. The cylindrical TE$_{011}$ cavity data were taken with the BDPA sample extending the entire cavity length and plotted in Fig. 6a. For a 120 ns $\pi$-pulse, the power was set to 2.5 W. In Fig. 6b, the UF re-entrant TE$_{\text {01U}}$ cavity with the BDPA sample extending the entire cavity length is shown. For a 120 ns $\pi$ pulse, the power was set to 5 W. A second sample with 9.5 mm length was centered in the UF re-entrant TE$_{\text {01U}}$ cavity region-of-interest. Experimental nutation data are plotted in Fig. 6c, and for a 120 ns $\pi$ pulse, the power was set to 5 W.

Nutation experiments show good results in terms of increased sensitivity and uniformity of the $B_1$ field. The nutations using the UF re-entrant TE$_{\text {01U}}$ cavity with the sample extending the entire length show clear improvements over the data from the cylindrical TE$_{011}$ cavity, as shown in Fig. 6a, b, respectively.

The UF re-entrant TE$_{\text {01U}}$ cavity with the 9.5 mm sample only in the region-of-interest, shown in Fig. 6c, has the nutations further extended and the initial off-set is further minimized. The Bruker E580 was only able to acquire 1000 ns of data, but with the 9.5 mm sample, there was signal seen as far as 1300 ns. Increasing the integration would give even better signal. These data show the advantages of uniform field cavities. Three differences of note are: (1) the nutations are improved by at least 40% and the initial off-set is reduced by 50%. (2) The first-order linear background subtraction is not adequate for the cylindrical TE$_{011}$ cavity. Higher order background exists and cannot be easily corrected. (3) A nutation signal phase inversion is exhibited in Fig. 6a at 1200 ns and another at 2400 ns, while only one inversion at 2800 ns is noticeable in Fig. 6b. We have experimentally attributed this oscillatory phase inversion to be due to inhomogeneity of the $B_1$ field, seemingly at the top and bottom of the resonator. The nutation signal phase inversion is shown to be minimized in Fig. 6c, but can be increased by moving the sample outside of the region-of-interest.

## Discussion

**Fig. 7:**
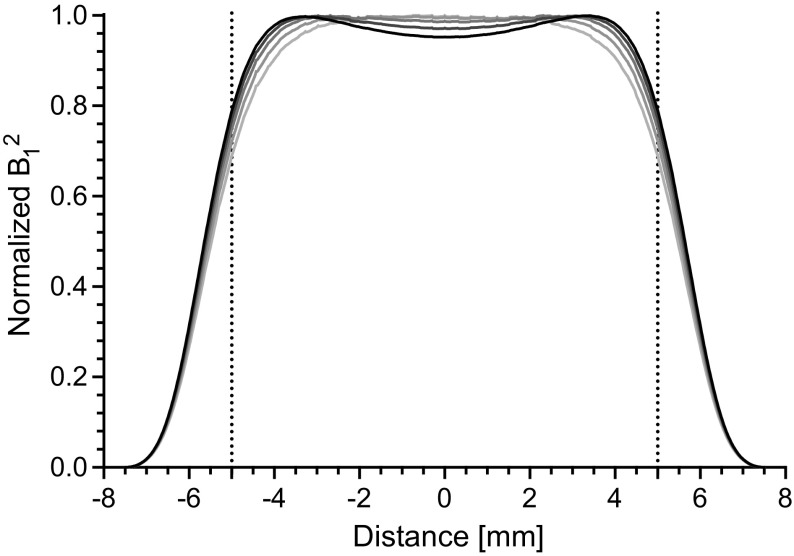
Ansys HFSS simulations of the microwave magnetic field squared (B$^2_1$) profile of the UF re-entrant cylindrical TE$_{\text {01U}}$ cavity for a range dielectrics. The dielectric constant, $\epsilon$, is varied from 1 to 5 (dark to light) with a fixed end-section geometry. Dotted lines mark the region-of-interest of the cylindrical re-entrant TE$_{\text {01U}}$ cavity. B$^2_1$ is used to highlight the differences and is proportional to EPR signal intensity

Dielectric loading variations due to different samples change the cut-off frequency of the region-of-interest and, thus, the uniformity condition of the resonator. Shown in Fig. 7 is the simulated microwave magnetic field squared ($B^2_1$) profile of the UF re-entrant cylindrical TE$_{\text {01U}}$ cavity for a range of dielectric constants ($\epsilon _r$ ranges from 1 to 5 in integer steps, with a loss tangent of 0.005) for a fixed Rexolite end-section geometry. $B^2_1$ is used to highlight the differences and is proportional to EPR signal intensity. The frequency shift due to the real part of the dielectric from 1 to 5 is 34.492, 34.206, 33.910, 33.592, and 33.264 GHz, respectively.

Although the resonator is designed for an $\epsilon _r$ of 3, good uniformity is exhibited for this limited range. This is an advantage of the UF re-entrant cavity compared to a UF cavity. Similar to a UF LGR, the electric field profile is more confined outside of the sample region and the $B_1$ field is stabilized by the current on the re-entrant fins. The uniformity over the entire sample volume varies from 80.1, 80.1, 79.8, 79.3, and 78.5%, as the dielectric is stepped from $\epsilon _r$ 1 to 5, respectively.

$\Lambda _\mathrm{ave}$ of the UF re-entrant TE$_{\text {01U}}$ resonator is lower by about 40% due to the $Q_0$ value being lower than expected. The lowering of the Q$_0$ value is due to the construction of the prototype resonator out of brass and higher losses in the Rexolite plastic than anticipated. Changing the resonator body to solid silver and experimenting with different plastics would be advantageous.

Higher stored energy in the coupling and iris region makes the system more frequency dependent. The frequency dependence of the H-type T-junction coupler without inductive perturbations was shown to have a large effect on the coupling efficiency, as shown in Fig. 4. In addition, by extending the iris over the entire length of the waveguide H-plane, a long-slot iris iss created [24]. The long-slot iris exhibits lower stored energy than a resonant iris or inductive hole and minimizes $B_1$ field perturbations. By splitting the long-slot iris to a dual-slot iris, the stored energy and frequency dependence is further reduced. In general, the reduction of stored energy outside of the resonator reduces the frequency dependence when tuning, matching, or changing samples. These design criteria are critical for UF resonators.

**Fig. 8:**
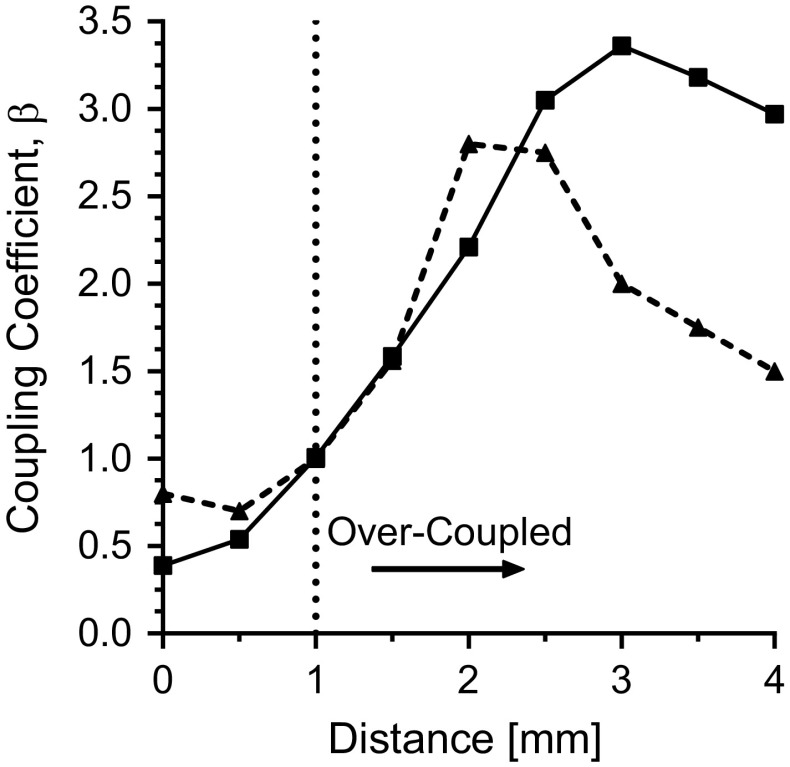
Bench measurements on the vector network analyzer of the coupling coefficient $\beta$ for the re-entrant TE$_{\text {01U}}$ cavity with H-type T-junction coupler and long-slot iris (solid) compared to the cylindrical TE$_{011}$ cavity with slot iris from Ref. [20] (dashed)

Shown in Fig. 8 are vector network analyzer measurements of the coupling coefficient $\beta$ for the re-entrant TE$_{\text {01U}}$ cavity with H-type T-junction coupler and long-slot iris (solid) compared to the cylindrical TE$_{011}$ cavity with slot iris from Ref. [20]. This shows better over-coupling performance for the re-entrant TE$_{\text {01U}}$ cavity which corresponds to a larger bandwidth by the equation:

5 $$\begin{aligned} Q_\mathrm{L} = \frac{Q_0}{\beta +1}, \end{aligned}$$

where the loaded *Q* value, $Q_\mathrm{L}$, is proportional to bandwidth by $1/\Delta f$ [10]. With a lower initial $Q_0$ value, the re-entrant TE$_{\text {01U}}$ cavity has a significant increase in bandwidth for comparable EPR signal. The re-entrant TE$_{\text {01U}}$ cavity has a calculated bandwidth of approximately 110 MHz, while the cylindrical TE$_{011}$ cavity in Ref. [20] has a calculated bandwidth of 46 MHz (Q$_0$ of 2400).

Finally, in the *x*- and *y*-directions, the B$_1$ field exhibits some variation. A smaller inner diameter capillary (with the same outer diameter) could be used to improve this variation, but will sacrifice EPR signal intensity. However, the *x*- and *y*-direction variation is already 15% better in a re-entrant geometry compared to the cylindrical TE$_{011}$ cavity from both a “sucking-in” effect of the quartz capillary and more confined electric field profile, as shown in Fig. 3. The capillary geometry of 2.8 mm OD and 1.8 mm ID was chosen to be compatible with our current standard Q-band capillary tubes.

## Conclusion

A uniform field re-entrant cylindrical TE$_{\text {01U}}$ cavity has been designed, fabricated, and tested to improve pulse EPR experiments. The microwave magnetic field, B$_1$, has been calculated and confirmed by measurements to be 88.3% uniform over the entire cavity and 98% uniform over the region-of-interest. By introducing re-entrant fins to a UF cylindrical TE$_{\text {01U}}$ cavity, the Q value of the re-entrant TE$_{\text {01U}}$ cavity is lowered, but the resonator efficiency and stored energy is increased. This new geometry yields similar signals as the standard cylindrical TE$_{011}$ while increasing $\Lambda _\mathrm{ave}$ by approximately 60%. The increase of $\Lambda _\mathrm{ave}$ affects pulse EPR experiments in two ways: (1) less microwave power is needed for the same tip angle and (2) the majority of the sample is excited at the same tip angle.

Initial results using a brass prototype resonator have shown significantly improved data quantified by the nutation experiments. In this work, we have shown that a UF re-entrant geometry can provide an enhanced efficiency parameter, increase EPR signal intensity, larger bandwidth, and a uniform microwave magnetic field along the sample volume to improve pulse EPR experiments. Future work includes a second generation resonator in solid silver, ESEEM, HYSCORE and ELDOR-detected NMR experiments (EDNMR), and extending the UF re-entrant TE$_{\text {01U}}$ cavity to W-band frequencies.
